# Bilateral Cerebral Mucormycosis in an Immunocompetent Female

**DOI:** 10.31486/toj.21.0088

**Published:** 2022

**Authors:** Remi T. Okwechime, Nicholas Reyes, Darshan Trivedi, Ifeanyi O. Iwuchukwu

**Affiliations:** ^1^ Department of Neurocritical Care, Ochsner Clinic Foundation, New Orleans, LA; ^2^ The University of Queensland Medical School, Ochsner Clinical School, New Orleans, LA; ^3^ Department of Pathology, Ochsner Clinic Foundation, New Orleans, LA

**Keywords:** *Central nervous system fungal infections*, *Mucorales*, *mucormycosis*

## Abstract

**Background:** Mucormycosis is a serious angioinvasive fungal infection. Immunocompromised patients are more likely to be susceptible to mucormycosis than immunocompetent individuals. Cerebral mucormycosis has been reported, but cases have primarily been unilateral. We report a case of bilateral cerebral mucormycosis in an immunocompetent patient.

**Case Report:** A 37-year-old female with no significant medical history was transferred to our tertiary center after cerebrospinal fluid profile following a lumbar puncture at an outside hospital suggested bacterial meningitis. Computed tomography of the head revealed hypodensity and cerebral edema in the left basal ganglia, and magnetic resonance imaging (MRI) brain showed increased T2 signal and mass-like configuration centered in the left basal ganglia. During her hospital stay, she had neurologic decompensation with respiratory failure. She was intubated and placed on mechanical ventilation. Repeat MRI brain revealed evolving cerebral edema signal and interval development of progression across the midline involving the right basal ganglia. Because of the aggressive nature of the lesion and cerebral edema, she underwent a biopsy with placement of an external ventricular drain. Despite medical and surgical interventions, she neurologically worsened and died. Histopathologic evaluation of the biopsied lesion revealed numerous fungal hyphae consistent with mucormycosis.

**Conclusion:** Our patient was not immunocompromised, and this case highlights the clinical challenges in initiating immunosuppressive therapy in a patient with rapidly progressive central nervous system disease.

## INTRODUCTION

Mucormycosis is a serious angioinvasive infection caused by fungi of the order of Mucorales.^1^ *Mucor* and *Rhizopus* are the principal genera that cause human disease, although others including *Cunninghamella*, *Absidia*, and *Apophysomyces* have also been isolated in infected individuals.^2^ Although fungal spores are ubiquitous, immunocompetent individuals are usually resistant to these pathogens. Immunocompromised patients with corticosteroid use, burns, hematologic malignancy, bone marrow or organ transplant, and diabetes mellitus are more likely to be susceptible to mucormycosis.^3^ Mucormycosis can have several presentations following the involvement of different organs, including pulmonary, gastrointestinal, cutaneous, renal, cerebral, rhino-orbital, and disseminated fungemia.^2^ Cases describing central nervous system (CNS) presentations of mucormycosis include an angioinvasive *Mucor* thrombus occluding the proximal segment of the internal carotid artery and causing an acute ischemic stroke.^3-9^ Ophthalmic artery occlusion and cavernous sinus thrombosis have also been described.^10,11^ While the majority of cases are unilateral, few bilateral cases have been reported in immunocompromised individuals or patients with diabetes mellitus.^12-15^ We report a case of bilateral cerebral mucormycosis with leptomeningeal involvement in an immunocompetent patient.

## CASE REPORT

A 37-year-old female presented to an outside hospital with the chief complaint of a headache that began 2 to 3 days prior to presentation. She had a medical history of mood disorder, hypertension, temporomandibular joint dysfunction, and chronic neck pain secondary to osteoarthritis with radiculopathy. At the initial presentation, she described the headache as waxing and waning in intensity, constant in timing, and with pain rated as moderate to severe. Her headache worsened with exposure to light, and she had associated fever, chills, neck stiffness, and generalized body aches. She developed right-sided weakness a few hours following presentation. She denied chest pain, shortness of breath, abdominal pain, rashes, nausea, vomiting, diarrhea, bleeding, or recent sick contacts. She reported having had 2 teeth removed approximately 1 week before presentation. She was noncompliant with her prescribed amoxicillin, having taken 10 of the prescribed 30 doses.

She reported a history of fever, mouth blisters, and genital herpes outbreaks and said she was treated for methicillin-resistant *Staphylococcus aureus* 7 weeks prior. She said she loved outdoor activities such as fishing but had not hiked or camped recently. The last time she swam in a chlorinated pool was 7 weeks prior. She worked in retail and had no prior use of steroids or history of recent travel. She was a 1 pack per day smoker and denied alcohol and intravenous (IV) drug use. Her surgical history was noncontributory, and she did not have a history of mucormycosis.

**Figure 1.**
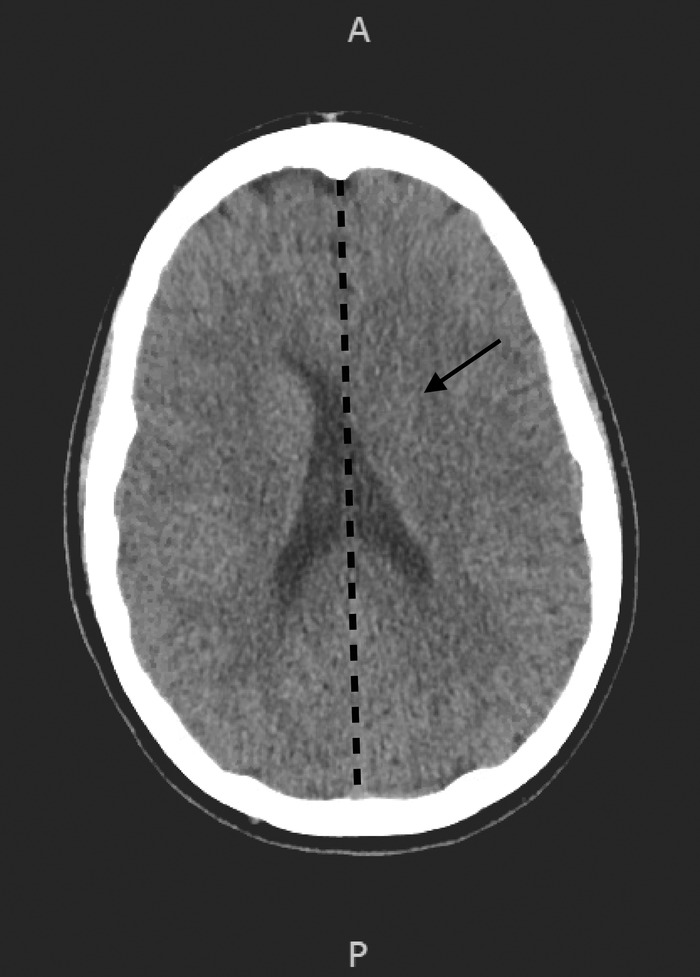
Computed tomography of the head without contrast upon arrival shows left basal ganglia hypodensity with mass effect on the left lateral ventricle (arrow) and 10.5-mm rightward shift of midline structures (midline depicted by dotted line).

**Figure 2.**
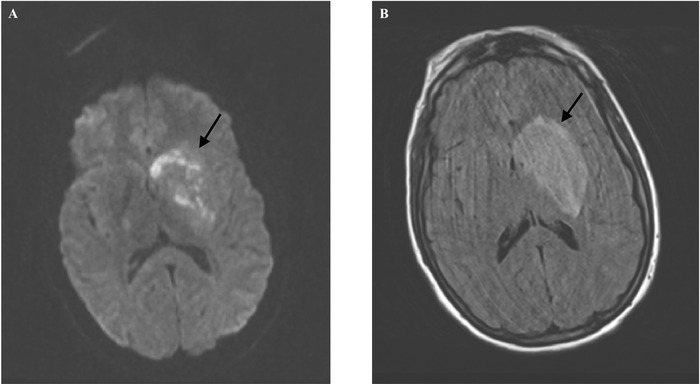
Mass-like lesion centered in the left basal ganglia extending to the left crus cerebri (arrows) is shown in (A) magnetic resonance imaging (MRI) brain diffusion weighted imaging (DWI) with contrast and (B) axial view MRI brain T2-weighted fluid-attenuated inversion recovery (FLAIR).

Initial investigations at presentation included a chest x-ray revealing clear lung fields and normal electrocardiogram. Computed tomography (CT) head without contrast revealed a left basal ganglia hypodensity with mass effect on the left lateral ventricle and 10.5-mm rightward shift of midline structures (Figure 1). Magnetic resonance imaging (MRI) brain with and without contrast showed a mass-like lesion centered in the left basal ganglia extending to the left crus cerebri (Figure 2). Transthoracic echocardiogram was normal with no evidence for thrombi or vegetations. HIV screening was negative. Complete blood count (CBC) showed a white blood cell count of 12.67 K/μL (reference range, 3.9-12.7 K/μL) with absolute neutrophil count of 10.7 K/μL (reference range, 1.8-7.7 K/μL). Cerebrospinal fluid (CSF) obtained via lumbar puncture was slightly hazy in appearance, and the results of the CSF study were as follows: protein of 101 mg/dL (reference range, 15-40 mg/dL), white blood cells of 1,457 mm^3^ (reference range, 0-5 mm^3^), neutrophils of 96%, (reference range, 0%-6%), and glucose of 49 mg/dL (reference range, 40-70 mg/dL). No organisms were seen on CSF gram stain or culture.

Given the patient's CSF findings and acuity of headache onset with accompanying fever and neck stiffness, bacterial meningitis was suspected. Her history of mouth blisters and genital herpes outbreaks provided evidence to include encephalitis in the differential diagnoses. Primary brain malignancy such as glioblastoma or astrocytoma was also suspected considering her CT head findings. Empiric antibiotics of IV vancomycin 1,250 mg every 12 hours and IV ceftriaxone 2 g every 12 hours, IV dexamethasone 4 mg every 6 hours, and IV acyclovir 10 mg/kg every 8 hours were started, and she was transferred to our tertiary center.

Following her transfer, physical examination showed neck stiffness, right hemiparesis, expressive aphasia, and new onset anisocoria that resolved with IV mannitol 50 g. Emergent CT head showed edema with a 3-mm rightward midline shift at the level of the foramen of Monro.

**Figure 3.**
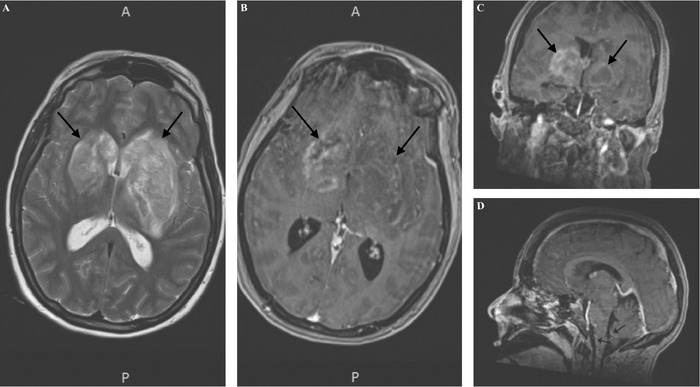
(A-C) Magnetic resonance imaging (MRI) brain with contrast shows peripheral enhancement of the basal ganglia lesion and new progression across the midline involving the right basal ganglia extending to the right cerebral peduncle (arrows). (D) MRI brain shows leptomeningeal enhancement of the superficial surface of the brainstem and proximal cervical spinal cord (arrows).

On day 2, the patient's neurologic condition declined, with worsening mental status and respiratory failure requiring endotracheal intubation with mechanical ventilation. Because of her rapid clinical decline, she underwent a follow-up MRI brain that showed peripheral enhancement of the basal ganglia lesion and new progression across the midline involving the right basal ganglia extending to the right cerebral peduncle. Leptomeningeal enhancement was also noted along the superficial surface of the brainstem and proximal cervical spinal cord (Figure 3).

**Figure 4.**
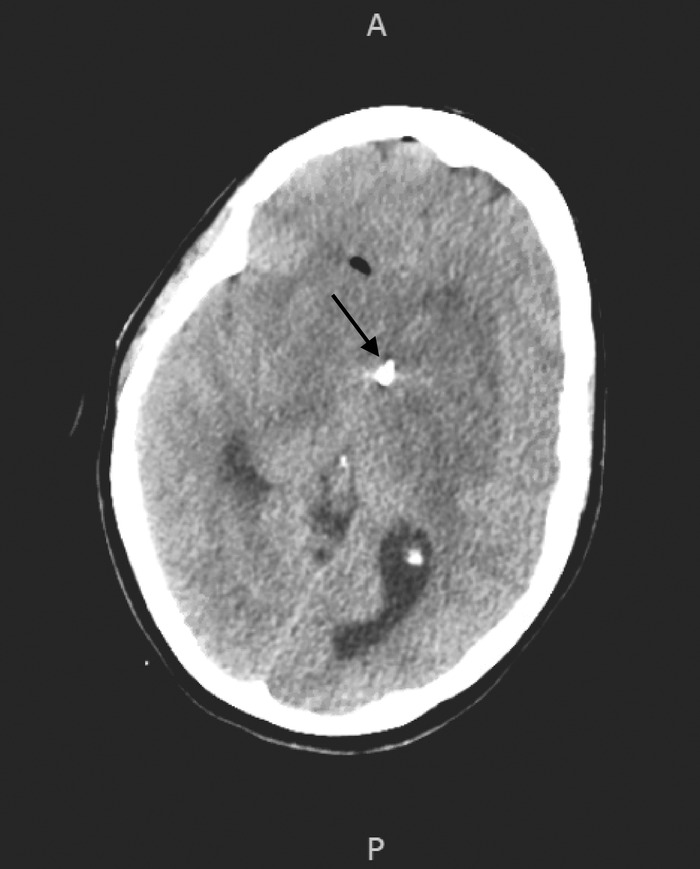
Computed tomography head after external ventricular drain placement (arrow).

Emergent neurosurgical evaluation was sought for a biopsy of the basal ganglia lesion, and an external ventricular drain was placed during the biopsy. Postoperative CT head did not show hydrocephalus (Figure 4). Notably, no pathologic masses or lesions were observed on CT body imaging on hospital day 2.

Repeat CBC showed an increase in white blood cell count to 14.05 K/μL, with absolute neutrophil count of 10.7 K/μL. Repeat CSF studies showed protein of 61 mg/dL, white blood cells of 243 mm^3^, neutrophils of 82%, and glucose of 69 mg/dL. IV ceftriaxone was changed to IV meropenem 2 g every 8 hours to cover CSF pathogens, anaerobes, and *Listeria*. IV acyclovir was discontinued because of low suspicion for herpes simplex virus encephalitis.

On day 3, episodes of elevated systolic blood pressures (>200 mm Hg) and intracranial pressures (ICP) as high as 30 mm Hg (reference range, <10-15 mm Hg) were associated with temperature spikes to 103.3 °F, bradycardia, roving eye movements, and bilateral upper and lower extremity spasticity with brisk reflexes. Repeat CBC showed increased white blood cell count of 14.29 K/μL, with absolute neutrophil count of 11.5 K/μL. Elevated blood pressure was managed with IV nicardipine 2.5 mg/hr with an additional single dose of IV hydralazine 10 mg and IV labetalol 10 mg. Bromocriptine 5 mg via orogastric tube every 8 hours and gabapentin 300 mg via orogastric tube every 8 hours were also added to her medication regimen for possible neurogenic dysautonomia. Because of the invasive pattern and rapid progression of the intracranial lesions, IV amphotericin B 5 mg/kg every 24 hours was added to cover fungal pathogens. A round of plasmapheresis was also attempted for autoimmune encephalitis, although the procedure was aborted after less than 30 minutes because of the patient's bradycardia and hypotension.

**Figure 5.**
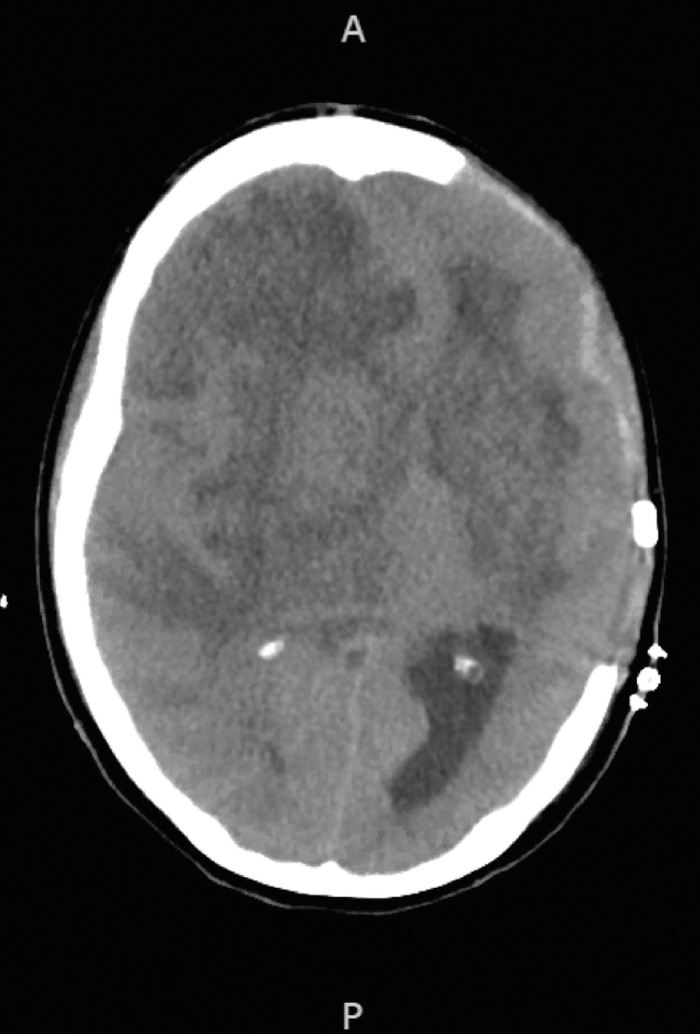
Computed tomography head after left decompressive hemicraniectomy.

On day 4, the patient experienced multiple ICP crises overnight (up to 49 mm Hg) associated with dysautonomia (temperature range 91.6-102.2 °F, pulse rate 34-99/min, systolic blood pressure 90-210 mm Hg, and diastolic blood pressure 49-96 mm Hg). She underwent a left decompressive hemicraniectomy because of rapidly progressive bilateral extensive zones of parenchymal edema (Figure 5).

Repeat CBC showed white blood cell count of 12.86 K/μL, with absolute neutrophil count of 11 K/μL. The possibility of an aggressive acute inflammatory demyelinating syndrome prompted the initiation of IV methylprednisolone 500 mg every 12 hours and a single dose of IV methotrexate 5,025 mg administered over 2 hours.

**Figure 6.**
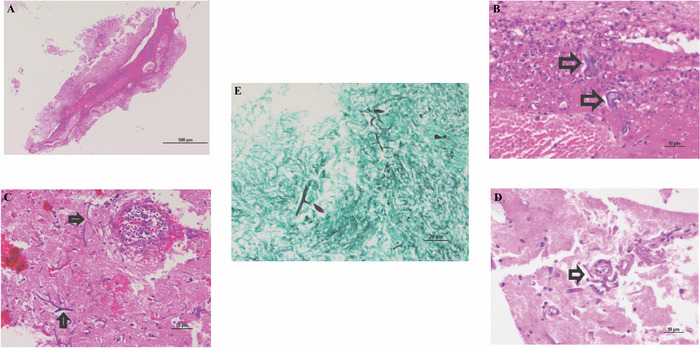
(A) At low power, an inflammatory process with acute hemorrhage is identified in the subarachnoid space, associated with the meningeal vessels (hematoxylin and eosin [H&E] stain, magnification ×40). (B) At higher power, the acute inflammatory process is seen associated with eosinophilic, broad, flat, ribbon-like forms, suggestive of fungal hyphae (arrows, H&E, magnification ×400). (C) The fungal hyphae demonstrate clear branching (arrows, H&E, magnification ×200). (D) Fungal hyphae can be seen associated with a blood vessel (arrow, H&E, magnification ×400). (E) Grocott methenamine silver special stain highlights the numerous viable fungal hyphae (magnification ×200).

On day 5, the patient's neurologic condition worsened with bilateral blown pupils and loss of brainstem reflexes. Repeat CBC showed white blood cell count of 10.79 K/μL, with absolute neutrophil count of 9.5 K/uL. On day 5, the patient died shortly after her family agreed to comfort measures. All the patient's bacterial blood and CSF cultures had been negative. Fungal cultures were not collected because of lack of follow-up. However, stereotactic brain biopsies on day 2 of admission and postmortem histopathology confirmed cerebral mucormycosis (Figure 6).

## DISCUSSION

Bilateral cerebral mucormycosis has been rarely documented but is usually life-threatening with multiple clinical presentations and is associated with IV drug abuse, decreased immunocompetency, or diabetes mellitus.^2^ Our patient did not have a notable route of infection. Ma et al examined 81 cases of mucormycosis worldwide and identified 11 clinical classification types according to the primary sites and invaded organs: rhinocerebral, rhino-orbital cerebral, ear-cerebral, oral-cerebral, meningoencephalitis, encephalitis, pulmonary, gastrointestinal, cutaneous, focus, and disseminated (also known as sepsis type).^16^ Of these classification types, the rhinocerebral and rhino-orbital cerebral mucormycosis types were the most common.^16,17^

Cerebral mucormycosis accounts for 9% of all cases of mucormycosis, and histopathology and culture are the diagnostic gold standard.^18,19^ Most reported cases have been unilateral. The route of infection and underlying immunity of the patient determine the outcome and clinical course.^19^ Even in the presence of risk factors such as IV drug use, the route of infection is not always clear.^20^ In Montgomery et al, an immunocompetent 36-year-old male with a history of IV substance abuse presented with isolated intracranial mucormycosis involving the right basal ganglia and associated cerebral edema.^20^ The presentation was uncommon because despite his known social risk factor of IV substance abuse, the patient had no evidence of expected cutaneous manifestations or rhinocerebral involvement.^20^ Multiple cases of cerebral mucormycosis have been reported after tooth extractions.^21^ Dental surgical trauma may compromise local vascularity and provide an entry port for the microorganisms. Angioinvasion of Mucorales and its spores leads to thrombosis and subsequent necrosis of associated hard and soft tissue.^21,22^ Our patient's cerebral mucormycosis may have been odontogenic in origin as she had teeth extracted approximately 1 week prior to presentation.

Cases of CNS mucormycosis with sino-orbital and rhinocerebral infections as the leading cause of invasive mucormycosis have been reported typically in diabetic immunocompromised hosts.^19^ Isolated cerebral mucormycosis has been reported as meningoencephalitis, as an abscess resulting from penetrating head trauma, and from hematogenous spread in patients who are IV drug users.^19^ Adler et al discussed an immunocompetent 24-year-old with a history of IV substance abuse who rapidly developed parkinsonism secondary to bilateral fungal abscesses (aspergillosis or mucormycosis).^23^

The medical management of CNS mucormycosis is primarily dependent on the rapid initiation of antifungals, particularly IV amphotericin B lipid formulation, with IV flucytosine or posaconazole commonly used for maintenance.^19^ However, the mortality rate is as high as 65% despite aggressive treatment measures.^19^ In addition to medical management, debridement of necrotic tissues and early biopsy with or without aggressive surgical resection can also be pursued.^19^ Alleyne et al described a radical surgical procedure of exenteration of the cavernous sinus, carotid sacrifice, and trigeminal nerve transection for a case of rhinocerebral mucormycosis.^24^ In comparison, Hamilton et al reported a more conservative multimodality treatment approach to rhinocerebral mucormycosis in a 14-year-old diabetic male that included antibiotics and hyperbaric oxygen following endoscopic sinus debridement of *Mucor* and necrotic bone and cerebral evacuation of abscesses.^25^ Ferguson et al suggest that higher partial pressures of oxygen have fungicidal properties and also serve as adjunctive treatment to oxygenate salvageable tissue.^26^ Because of the diagnostic dilemma in the early stages of our patient's case, treatment with immunosuppressive therapies may have contributed to the rapid progression and aggressiveness of the CNS mucormycosis.

An ICP crisis in our patient occurred a few days after brain biopsy, demonstrating rapid progression of the disease confirmed by follow-up neuroimaging studies. Dhakar et al speculate that imaging may be the only clue in identifying this diagnostically challenging disease.^12^ Uncertainties and lack of early diagnostic tools cause delays in initiating appropriate antifungal treatment which likely contribute to poor outcomes in mucormycosis.^20^ Molecular diagnostic methods are likely more effective than tissue biopsy and histopathology in the identification of Mucorales and may be useful in the diagnosis of microscopic pathogens.^27^ Zhang et al emphasize the application of next-generation sequencing technology in early stages of intracranial mucormycosis to provide earlier identification and therefore improve treatment and prognosis for patients,^28^ showing that other investigative methods may prove difficult (the positive culture rate is only 50% for all types of mucormycosis with a confirmed pathologic diagnosis and only 38% for cerebral mucormycosis).^2,19^ The diagnostic gold standard of tissue biopsy and histopathology is not always feasible, particularly in vulnerable populations.^18^

## CONCLUSION

Cerebral mucormycosis is a rare infection that is rapidly fatal, especially when not identified early. It most commonly affects immunocompromised patients and individuals with diabetes mellitus but may present in an immunocompetent patient. Our patient was not immunocompromised, and the case highlights the challenges of timely diagnosis and management in a rapidly progressive CNS disease.

## Notes

*This article meets the Accreditation Council for Graduate Medical Education and the American Board of Medical Specialties Maintenance of Certification competencies for Patient Care and Medical Knowledge.*
